# Implications of different approaches for characterizing ambient air pollutant concentrations within the urban airshed for time-series studies and health benefits analyses

**DOI:** 10.1186/1476-069X-10-36

**Published:** 2011-05-11

**Authors:** Matthew J Strickland, Lyndsey A Darrow, James A Mulholland, Mitchel Klein, W Dana Flanders, Andrea Winquist, Paige E Tolbert

**Affiliations:** 1 Department of Environmental Health, Rollins School of Public Health, Emory University, Atlanta, Georgia, USA; 2 Department of Epidemiology, Rollins School of Public Health, Emory University, Atlanta, Georgia, USA; 3 Department of Civil and Environmental Engineering, Georgia Institute of Technology, Atlanta, Georgia, USA

## Abstract

**Background:**

In time-series studies of the health effects of urban air pollutants, decisions must be made about how to characterize pollutant levels within the airshed.

**Methods:**

Emergency department visits for pediatric asthma exacerbations were collected from Atlanta hospitals. Concentrations of carbon monoxide, nitrogen dioxide, ozone, sulfur dioxide, particulate matter less than 10 microns in diameter (PM_10_), particulate matter less than 2.5 microns in diameter (PM_2.5_), and the PM_2.5 _components elemental carbon, organic carbon, and sulfate were obtained from networks of ambient air quality monitors. For each pollutant we created three different daily metrics. For one metric we used the measurements from a centrally-located monitor; for the second we averaged measurements across the network of monitors; and for the third we estimated the population-weighted average concentration using an isotropic spatial model. Rate ratios for each of the metrics were estimated from time-series models.

**Results:**

For pollutants with relatively homogeneous spatial distributions we observed only small differences in the rate ratio across the three metrics. Conversely, for spatially heterogeneous pollutants we observed larger differences in the rate ratios. For a given pollutant, the strength of evidence for an association (i.e., chi-square statistics) tended to be similar across metrics.

**Conclusions:**

Given that the chi-square statistics were similar across the metrics, the differences in the rate ratios for the spatially heterogeneous pollutants may seem like a relatively small issue. However, these differences are important for health benefits analyses, where results from epidemiological studies on the health effects of pollutants (per unit change in concentration) are used to predict the health impacts of a reduction in pollutant concentrations. We discuss the relative merits of the different metrics as they pertain to time-series studies and health benefits analyses.

## Background

The adoption, evaluation, and revision of ambient air quality standards are dynamic processes. Results from epidemiological studies on the health effects of ambient air pollutant concentrations figure prominently in these processes. In synthesizing the body of scientific work to inform policy, a major goal is to describe the concentration-response relationships between ambient air pollutant concentrations and various health outcomes. Synthesis of concentration-response associations, however, is complicated not only by city-to-city differences, but also by methodological differences across studies. As the field of air pollution epidemiology has grown, so has the breadth and complexity of the literature, which now encompasses many different study designs, methods to estimate and assign exposure, and statistical approaches [1,2].

One fundamental issue for time-series studies is how to characterize measurements of ambient air pollutant levels within the airshed; indeed, even in our ongoing Study of Particles and Health in Atlanta (SOPHIA), in which we have been investigating the short-term effects of ambient air pollutant concentrations on a broad range of health outcomes, we have at times characterized pollutant concentrations using measurements from a centrally-located monitoring station [3-5], and at other times have averaged concentrations across monitors using population-weighting [6,7]. In addition to these, a commonly used approach in the literature is to average the measurements across the network of monitors (ignoring population density) [8]. Although the time series that result from these approaches may be well-correlated, the distributions of pollutant concentrations may differ, and these differences can affect the concentration-response estimates from the epidemiologic models. One way to compensate for these differences is to report the effect estimates per interquartile range (IQR) increase, as opposed to per unit increase (e.g., per 10 μg/m^3 ^increase). However, rescaling the estimates in this manner has the disadvantage of tying the interpretation of the effect estimate to the distribution of the pollutant metric used in that study.

For risk assessment and health benefits analyses the effect estimates reported in the literature are often converted into changes per one-unit increase in pollutant concentration. For example, the US EPA's BenMAP software, which is used to estimate health and economic impacts for a change in air quality, contains a library of concentration-response functions from epidemiological studies in which the effect estimates have all been rescaled to unit increases in concentration [9]. The process of rescaling the estimates from the various studies into a common unit has the appearance of increasing the comparability of the estimates across studies, because the effect estimates now correspond to the same change in concentration. And while most would agree that this rescaling is necessary, between-study differences in the approach used to characterize air pollution levels within the airshed may be a source of heterogeneity among the estimates.

Using data from the SOPHIA study, we present three different approaches for summarizing daily ambient air pollutant concentrations within the Atlanta airshed, and we evaluate how the pollutant metrics produced from these approaches impact estimates of concentration-response. We argue that the metrics are estimates of different underlying quantities, and therefore heterogeneity in the concentration-response estimates (per unit increase) across the metrics should be expected. Rather than view any particular metric as "optimal" in a broad context, we suggest that the appropriateness of a given metric will depend on the research or policy question of interest.

## Methods

The main results from our time-series investigation of associations between population-weighted average ambient air pollutant concentrations and emergency department visits for pediatric asthma exacerbations in metropolitan Atlanta during 1993-2004 have been reported elsewhere [7]. For the present analysis we used the same outcome, which we defined as all emergency department visits with an ICD-9 code for either asthma (493.0-493.9) or wheeze (786.09 before October 1, 1998; 786.07 on and after October 1, 1998) among children 5-17 years of age.

Measurements of ambient air pollutant concentrations were obtained from three networks of stationary monitors in Atlanta: the EPA Air Quality System, including State and Local Air Monitoring System and Speciation Trends Network for PM2.5 component measures; the Southeastern Aerosol Research and Characterization Study [10], including the Atlanta EPA supersite at Jefferson Street [11]; and the Assessment of Spatial Aerosol Composition in Atlanta network [12]. For the present analysis we investigated nine different pollutants, measured as: one-hour maximum carbon monoxide, nitrogen dioxide, and sulfur dioxide; eight-hour maximum ozone; and 24-hour average particulate matter less than 10 microns in aerodynamic diameter (PM_10_), particulate matter less than 2.5 microns in aerodynamic diameter (PM_2.5_), and the PM_2.5 _components sulfate, organic carbon, and elemental carbon. Each pollutant was measured by at least three different stationary monitoring stations. We obtained daily measurements during 1993 through 2004 for carbon monoxide, nitrogen dioxide, ozone, and sulfur dioxide; during 1996 through 2004 for PM_10_; and during August 1, 1998 through 2004 for PM_2.5 _and the various PM_2.5 _components. We selected one downtown monitor to be the "central monitor" for each pollutant.

For each pollutant we created three different daily metrics for use in the time-series analysis. Measurements from the central monitor were used for one metric. The second metric was the unweighted average of measurements from the network of monitoring stations. To calculate this metric, the measurements at each monitor were log-transformed (because concentrations were right-skewed) and standardized using the mean and standard deviation at that monitor. These standardized values were averaged across monitors, unstandardized (using the overall mean and standard deviation), and exponentiated [8]. For the third metric, the population-weighted average concentration, we created statistical models to characterize the spatial variability of ambient air pollutant concentrations throughout Atlanta [13,14]. At each monitor the measurements were log-transformed and then standardized using the mean and standard deviation at that monitor. Daily surfaces were created by inverse distance-square weighting the standardized values. The daily pollutant concentrations at each Census tract centroid within 20-county metropolitan Atlanta (an area covering 16,079 square kilometers (6,208 square miles)) were estimated by converting the standardized value back to a concentration using an isotropic model that relates the means and standard deviations of the concentrations to the distance between the centroid and the urban center. Model diagnostics are available [14]. On each day we calculated the population-weighted average by weighting the estimated pollutant concentration at each Census tract by the number of people residing in that tract. Thus, for each pollutant, we created three daily metrics: one based on central monitoring station measurements, one based on averaging measurements across monitors, and one based on population-weighting the Census tract estimates from the spatial model. To ensure the same days were represented across the three metrics we restricted analysis to days when the central monitoring station had a valid measurement.

We implemented Poisson time-series regression models that accounted for overdispersion [15] to investigate short-term associations between emergency department visits for pediatric asthma and the various metrics. We limited our analysis to the Atlanta "warm season" (May through October), because in our previous work we observed several positive and statistically significant effects during the warm season [7], and our present goal is to evaluate the extent to which the different metrics impact estimates of concentration-response. The outcome variable was the hospital-specific daily count of pediatric asthma emergency department visits, and pollutant concentrations were characterized as three-day moving averages (average of concentrations today [lag 0], yesterday [lag 1], and the day before yesterday [lag 2]). All models contained indicator variables for lag 0 maximum temperature (for each degree Celsius), day of week, year, month, and hospital; cubic polynomials for day of season, lag 0-2 average dew point, and lag 1-2 average minimum temperature; and interactions between month and year, month and lag 0 maximum temperature, and month and day of week. We also controlled for the logarithm of the daily count of emergency department visits for acute respiratory infections among children age 5-17 years (excluding those who also had asthma or wheeze), as we observed that this was a strong predictor of the daily count of asthma emergency department visits [7]. Rate ratios from each model are presented per unit increase and per IQR increase, with the IQR defined according to the distribution of concentrations specific to the metric. Chi-square statistics, p-values, and 95%CI are provided to facilitate comparisons across the different approaches.

## Results

During the warm seasons of 1993-2004 we identified 41,741 emergency department visits for asthma or wheeze among children age 5-17 years. Descriptive statistics for the nine pollutants investigated are presented in Table 1. For all the pollutants except PM_2.5 _organic carbon, the mean at the central monitoring station was higher than the mean of the population-weighted average, reflecting the tendency for pollutants to be high near the urban core. The IQRs followed a similar pattern, where (save for PM_2.5 _organic carbon) there was more variability at the central monitor than there was for the population-weighted average. For the unweighted average concentration, some of the means and IQRs were similar to those at the central monitor, whereas others more closely resembled those from the population-weighted average. Spearman correlation coefficients for the three metrics, by pollutant, are presented in Table 2. These correlations were high for all pollutants examined, ranging from 0.99 to 0.80. Thus, even though the means and IQRs for a given pollutant may have differed across metrics, the metrics were all well-correlated over time.

**Table 1:** Descriptive statistics of ambient air pollutant concentrations in metropolitan Atlanta during May through October.^a^

Pollutant	Number of Monitors	Number of Observations	Central monitor Mean (IQR)	Unweighted average Mean (IQR)	Population-weighted average Mean (IQR)
8-h ozone, ppb^b^	5	2205	56.7 (26.4)	54.8 (25.0)	55.3 (22.6)

24-h PM_10_, μg/m^3c^	9	1563	29.4 (13.4)	29.7 (14.0)	27.4 (12.4)

24-h PM_2.5_, μg/m^3d^	11	1134	19.2 (9.8)	19.9 (9.1)	18.4 (8.7)

24-h PM_2.5 _sulfate, μg/m^3d^	6	980	6.3 (3.7)	5.8 (3.6)	5.8 (3.5)

24-h PM_2.5 _organic carbon, μg/m^3d^	6	1089	4.19 (1.92)	4.60 (2.45)	4.71 (2.71)

24-h PM_2.5 _elemental carbon, μg/m^3d^	6	1089	1.62 (0.91)	1.25 (0.68)	0.82 (0.53)

1-h nitrogen dioxide, ppb^b^	6	2190	42.0 (19.7)	27.7 (13.5)	22.0 (10.1)

1-h carbon monoxide, ppm^b^	3	1999	1.45 (0.83)	1.39 (0.92)	0.87 (0.46)

1-h sulfur dioxide, ppb^b^	5	2201	13.9 (13.0)	10.2 (7.7)	9.6 (7.6)

Abbreviations: interquartile range (IQR), hour (h), parts per billion (ppb), micrograms per cubic meter (μg/m^3^), parts per million (ppm).

^a^Ambient air pollutant concentrations are presented as three-day moving averages.

^b^Data begin on May 1, 1993.

^c^Data begin on May 1, 1996.

^d^Data begin on August 1, 1998.

**Table 2:** Pairwise Spearman correlation coefficients for three-day moving average ambient air pollutant concentrations.

	Correlation between central monitor and unweighted average	Correlation between central monitor and population-weighted average	Correlation between population-weighted average and unweighted average
8-h ozone	0.988	0.979	0.988

24-h PM_10_	0.956	0.961	0.990

24-h PM_2.5_	0.969	0.963	0.995

24-h PM_2.5 _sulfate	0.938	0.957	0.989

24-h PM_2.5 _organic carbon	0.847	0.891	0.975

24-h PM_2.5 _elemental carbon	0.831	0.804	0.966

1-h nitrogen dioxide	0.831	0.868	0.919

1-h carbon monoxide	0.960	0.946	0.980

1-h sulfur dioxide	0.849	0.917	0.954

Abbreviations: hour (h).

All correlation coefficients were statistically significant (p < 0.001).

Rate ratios for the associations between pediatric asthma emergency department visits and the pollution metrics, scaled to unit increases in concentration, are presented in Table 3. For the pollutants that had similar average concentrations across the metrics - ozone, PM_10_, PM_2.5_, PM_2.5 _sulfate, and PM_2.5 _organic carbon - the rate ratios and 95%CI per unit increases were also similar. For the pollutants with larger differences in average concentrations - PM_2.5 _elemental carbon, nitrogen dioxide, carbon monoxide, and sulfur dioxide - the rate ratios per unit increases varied across metrics. As displayed in Table 4, once the rate ratios were scaled to IQR increases in concentration, much of this heterogeneity went away. Scaling the rate ratios to an IQR increase is somewhat analogous to standardizing the distributions of the pollutant concentrations. The similarity of the rate ratios per IQR increase was due to the high temporal correlations among the metrics (Table 2). Although this pattern held for most of the pollutants examined, sulfur dioxide and PM_2.5 _organic carbon were exceptions. For these two pollutants the chi-square statistics and p-values varied appreciably across the metrics, with the unweighted average having the largest chi-square statistic in both instances. Here the rate ratios per IQR increase were less similar because the metrics varied in their ability to predict the health outcome.

**Table 3:** Associations between pollutant concentrations and pediatric asthma emergency department visits per unit increases in concentration.^a^

	Central monitor		Unweighted average		Population-weighted average	
Pollutant	RR, 95% CI	Chi-square, p-value	RR, 95% CI	Chi-square, p-value	RR, 95% CI	Chi-square, p-value
8-h ozone, per 25 ppb	1.061 (1.033, 1.090)	χ^2 ^= 18.89, p < 0.001	1.065 (1.036, 1.096)	χ^2 ^= 19.47, p < 0.001	1.070 (1.037, 1.104)	χ^2 ^= 17.68, p < 0.001

24-h PM_10_, per 10 μg/m^3^	1.018 (1.002, 1.034)	χ^2 ^= 4.82, p = 0.028	1.017 (1.001, 1.033)	χ^2 ^= 4.40, p = 0.036	1.016 (0.999, 1.033)	χ^2 ^= 3.31, p = 0.069

24-h PM_2.5_, per 10 μg/m^3^	1.031 (1.006, 1.057)	χ^2 ^= 6.08, p = 0.014	1.042 (1.014, 1.071)	χ^2 ^= 8.96, p = 0.003	1.044 (1.014, 1.075)	χ^2 ^= 8.28, p = 0.004

24-h PM_2.5 _sulfate, per 5 μg/m^3^	1.035 (1.003, 1.069)	χ^2 ^= 4.63, p = 0.031	1.042 (1.007, 1.077)	χ^2 ^= 5.64, p = 0.018	1.040 (1.005, 1.077)	χ^2 ^= 4.93, p = 0.026

24-h PM_2.5 _organic carbon, per 2 μg/m^3^	1.022 (0.997, 1.047)	χ^2 ^= 3.05, p = 0.081	1.031 (1.010, 1.053)	χ^2 ^= 8.39, p = 0.004	1.021 (1.003, 1.040)	χ^2 ^= 5.43, p = 0.020

24-h PM_2.5 _elemental carbon, per 1 μg/m^3^	1.028 (1.006, 1.051)	χ^2 ^= 6.22, p = 0.013	1.036 (1.008, 1.065)	χ^2 ^= 6.55, p = 0.010	1.065 (1.016, 1.116)	χ^2 ^= 6.97, p = 0.008

1-h nitrogen dioxide, per 20 ppb	1.052 (1.028, 1.077)	χ^2 ^= 17.98, p < 0.001	1.079 (1.044, 1.116)	χ^2 ^= 19.81, p < 0.001	1.105 (1.060, 1.152)	χ^2 ^= 22.30, p < 0.001

1-h carbon monoxide, per 1 ppm	1.044 (1.019, 1.070)	χ^2 ^= 12.71, p < 0.001	1.042 (1.015, 1.068)	χ^2 ^= 9.80, p = 0.002	1.096 (1.039, 1.156)	χ^2 ^= 11.32, p = 0.001

1-h sulfur dioxide, per 20 ppb	1.015 (0.998, 1.041)	χ^2 ^= 1.18, p = 0.277	1.062 (1.014, 1.111)	χ^2 ^= 6.60, p = 0.010	1.053 (1.004, 1.104)	χ^2 ^= 4.48, p = 0.034

Abbreviations: interquartile range (IQR), rate ratio (RR), confidence interval (CI), hour (h), parts per billion (ppb), micrograms per cubic meter (μg/m^3^), parts per million (ppm).

^a^Rate ratios are for three-day moving average (average of lag 0, lag 1, and lag 2) pollutant concentrations during the six warmest months in Atlanta (May through October).

**Table 4:** Associations between pollutant concentrations and pediatric asthma emergency department visits per IQR increases in concentration.^a^

	Central monitor		Unweighted average		Population-weighted average	
Pollutant	RR, 95% CI	Chi-square, p-value	RR, 95% CI	Chi-square, p-value	RR, 95% CI	Chi-square, p-value
8-h ozone, per IQR	1.065 (1.035, 1.095)	χ^2 ^= 18.89, p < 0.001	1.065 (1.036, 1.095)	χ^2 ^= 19.47, p < 0.001	1.063 (1.033, 1.094)	χ^2 ^= 17.68, p < 0.001

24-h PM_10_, per IQR	1.024 (1.003, 1.046)	χ^2 ^= 4.82, p = 0.028	1.024 (1.002, 1.046)	χ^2 ^= 4.40, p = 0.036	1.020 (0.998, 1.041)	χ^2 ^= 3.31, p = 0.069

24-h PM_2.5_, per IQR	1.031 (1.006, 1.056)	χ^2 ^= 6.08, p = 0.014	1.039 (1.013, 1.065)	χ^2 ^= 8.96, p = 0.003	1.038 (1.012, 1.065)	χ^2 ^= 8.28, p = 0.004

24-h PM_2.5 _sulfate, per IQR	1.026 (1.002, 1.051)	χ^2 ^= 4.63, p = 0.031	1.030 (1.005, 1.056)	χ^2 ^= 5.64, p = 0.018	1.028 (1.003, 1.053)	χ^2 ^= 4.93, p = 0.026

24-h PM_2.5 _organic carbon, per IQR	1.021 (0.997, 1.045)	χ^2 ^= 3.05, p = 0.081	1.038 (1.012, 1.065)	χ^2 ^= 8.39, p = 0.004	1.029 (1.005, 1.054)	χ^2 ^= 5.43, p = 0.020

24-h PM_2.5 _elemental carbon, per IQR	1.026 (1.005, 1.047)	χ^2 ^= 6.22, p = 0.013	1.025 (1.006, 1.044)	χ^2 ^= 6.55, p = 0.010	1.034 (1.009, 1.060)	χ^2 ^= 6.97, p = 0.008

1-h nitrogen dioxide, per IQR	1.051 (1.027, 1.076)	χ^2 ^= 17.98, p < 0.001	1.053 (1.029, 1.077)	χ^2 ^= 19.81, p < 0.001	1.052 (1.030, 1.074)	χ^2 ^= 22.30, p < 0.001

1-h carbon monoxide, per IQR	1.037 (1.016, 1.058)	χ^2 ^= 12.17, p < 0.001	1.038 (1.014, 1.063)	χ^2 ^= 9.80, p = 0.002	1.043 (1.018, 1.069)	χ^2 ^= 11.32, p = 0.001

1-h sulfur dioxide, per IQR	1.009 (0.992, 1.027)	χ^2 ^= 1.18, p = 0.277	1.023 (1.006, 1.042)	χ^2 ^= 6.60, p = 0.010	1.020 (1.001, 1.039)	χ^2 ^= 4.48, p = 0.034

Abbreviations: interquartile range (IQR), rate ratio (RR), confidence interval (CI), hour (h), parts per billion (ppb), micrograms per cubic meter (μg/m^3^), parts per million (ppm).

^a^Rate ratios are for three-day moving average (average of lag 0, lag 1, and lag 2) pollutant concentrations during the six warmest months in Atlanta (May through October).

## Discussion

Our analyses demonstrate how the method used to characterize ambient air pollutant concentrations in time-series studies can impact estimates of concentration-response. Because the spatial distribution of pollutant concentrations was similar from one day to the next the three metrics were well-correlated over time; consequently the chi-square statistics from the time-series models were similar across metrics. The rate ratios per unit increase were comparable across the metrics for pollutants with relatively homogeneous spatial distributions, whereas we observed larger differences for the pollutants with heterogeneous spatial distributions. Our findings lend support to the conclusions recently made by Peng and Bell [16] on the impacts of spatial misalignment in time-series studies.

The high temporal correlation among the metrics may give the impression that all three are estimates of the same underlying quantity. Each is picking up the same temporal signal (which is presumably a function of the total amount of pollution in the airshed), and thus the differences among the metrics appears to be an issue of calibration. For time-series investigations, in which interest typically centers on whether or not there is evidence for an association between air pollution levels and the rate of disease (i.e., the chi-square statistic and accompanying p-value), the differences among the metrics may seem relatively unimportant because all provide similar evidence for an association. The common practice of reporting results per IQR increase would seem to support this line of thinking - that it is the strength of evidence for the association, rather than the rate ratio per unit increase, that is of primary interest. However, when epidemiological results are used in health benefits analyses, such as those that have been conducted by the U.S. EPA [17,18] and others [19-23], the health effects of air pollution per unit increase in concentration become of central importance. The analyst must decide which concentration-response function(s) to use in the health benefits analysis, and as our results indicate, for pollutants with substantial spatial heterogeneity the choice of metric can meaningfully impact the rate ratio.

A natural question is to ask which of the metrics that we examined (if any) is the preferred approach for summarizing pollutant concentrations in a time-series study. Our suggestion is for investigators to allow the research or policy question of interest to guide the choice of metric. For example, the U.S. EPA annually examines each monitor to determine whether that site is in compliance with the National Ambient Air Quality Standards (NAAQS). Although there may be several monitors within an urban area, only one monitor has to be in violation of the NAAQS for the area to be considered out of attainment. Given that compliance with the NAAQS is based on measurements from the highest monitor (which is often located near the urban core), one might be interested in estimating the health benefits that would have been attained had that central monitor been in compliance. If this is the motivating public health policy question, then we believe it is appropriate to use the measurements from a centrally located monitor as the metric in a time-series study, and to use the rate ratio that results from that analysis in a health benefits analysis. For this health benefits estimate to be valid, the relationship between the concentrations at the central monitor and the rest of the airshed must be similar for both the observed (baseline) and alternative (policy/intervention) scenarios. Whether this assumption is met will largely depend on the intervention under consideration. For a widespread intervention this assumption is likely to be reasonable, whereas it may be very poor if emission controls are only applied locally near the central monitor.

Alternatively, the research or policy question of interest might center on the health effects of personal exposure to outdoor air pollutants. For example, one might want to estimate the health benefits that would have been achieved had each individual's exposure to ambient PM_2.5 _been 1 μg/m^3 ^lower. A good metric for addressing this question would be the average of the time- and location-weighted ambient air pollutant concentrations for each individual in the population. The error resulting from this metric would be expected to be predominantly of the Berkson type and would not substantially bias estimates of concentration-response [24-26]. Both the population-weighted average and the unweighted average can be viewed as surrogates of this metric, and therefore a health benefits analysis aimed at addressing this policy question would want to use a rate ratio based on one of these two metrics (as opposed to central monitor measurements). Of these two metrics, the population-weighted average seems like it should be the better surrogate because the pollutant concentrations are weighted based on residence, an approximation that is probably better for retirees and children, who likely spend a substantial amount of time near their home, than it is for working-age adults who are more likely to be commuting into the city. Even so, this metric did not systematically have larger chi-square statistics than the unweighted average, so it is difficult to argue that our results empirically support the population-weighted metric over the unweighted average. Further, the population-weighted average is a model-based estimate, and all models are misspecified to some degree. We did not attempt to incorporate the uncertainty from the modeled estimates into the analyses, although approaches for doing so have been proposed [27]. How well the unweighted average approximates the average of the individual-level time- and location-weighted ambient air pollutant concentrations is difficult to evaluate because the spatial distribution of monitoring stations differed by pollutant. For example, whereas the majority of the PM_2.5 _elemental carbon monitors were located near the urban core, the NO_2 _monitors were more uniformly distributed throughout the study area. Consequently, the unweighted average is (in effect) more heavily weighted towards downtown for PM_2.5 _elemental carbon than for NO_2_.

Although our results are based on air quality measurements from Atlanta, we expect that our findings have generalizability, since the basic features of the spatial distribution of pollutants are likely to be similar in other areas. Primary pollutants tend to have substantial spatial variability, and the sources of these pollutants (e.g., traffic) are often concentrated near the urban core. For these pollutants, the measurements from monitors near the sources will usually be higher than the measurements from monitors located farther away from the sources. Conversely, pollutants of secondary origin tend to be more widely dispersed throughout the urban airshed, such that measurements at the downtown monitors will often be similar to the measurements at monitors near the periphery. The magnitude of the differences in the rate ratios that we observed across the three metrics, however, is probably specific to our study. In particular, our study is based on a fairly large and mostly flat geographic area (20-county Atlanta). If we had chosen a smaller region then the differences among the three rate ratios per unit increase would have been smaller [28]. These differences were also likely affected by the choice of lag period, as a shorter lag period (e.g., one-day) would tend to increase the differences across metrics whereas a longer lag period would tend to lessen the differences. If Atlanta had significant geophysical landscape characteristics (e.g., mountains, valleys, or coastlines) then these could have affected the results as well.

The differing chi-square statistics from the time-series models for sulfur dioxide (χ^2 ^= 1.18 for the central monitor, χ^2 ^= 4.48 for the population-weighted average, and χ^2 ^= 6.60 for the uweighted average) and for PM_2.5 _organic carbon (χ^2 ^= 3.05 for the central monitor, χ^2 ^= 5.43 for the population-weighted average, and χ^2 ^= 8.39 for the uweighted average) were findings we did not expect a priori. These findings may point to the uncertainty that is present in the characterization of sulfur dioxide and PM_2.5 _organic carbon and to the inability of a central measurement to capture the spatially heterogeneous surfaces of these pollutants. In Atlanta, sulfur dioxide has only a few point sources (coal-burning power plants), and the measurements are strongly impacted by plume touchdowns. Consequently, the central monitoring station captures high events at the center of the city but misses plume touch downs that occur in other parts of the city. With respect to PM_2.5 _organic carbon, we know that particles of both primary and secondary origin are present in the airshed, and that the fraction of particles of primary origin tends to be greater near the urban core. As there is some evidence to suggest that the respiratory health effects of primary organic aerosols may differ from those of secondary organic aerosols [29], it is possible that the differences we observed for PM_2.5 _organic carbon are due to the relative toxicity of primary vs. secondary particles. There may be instrument error issues as well, and averaging measurements across monitors may help to dampen this source of measurement error [16,26,30].

Our findings are similar to those recently reported by Zauli Sajani et al. [31], who describe a set of "counterintuitive results" obtained from a case-crossover analysis of short-term associations between ambient air pollutant concentrations and mortality in the Emilia Romagna region of Italy. In that report the authors demonstrate how the estimated odds ratios (per 10 μg/m^3 ^increase) gradually increased as they enlarged the study area, noting that the variability in pollutant concentrations decreased with increasing aggregation. The authors hypothesized that measurement error might explain their findings, suggesting that "larger aggregation improves the representativity of the exposure estimates by decreasing exposure misclassification, which is more profound when using individual stations vs. regional averages" [31]. In Atlanta we also observed that the rate ratios increased (per unit increase in pollution) as we moved from central monitoring station measurements towards an average of measurements across monitors. In our data, however, we saw little indication that a reduction in measurement error was responsible for this pattern, as the chi-square statistics tended to be similar across the metrics. Instead, we believe that an alternative explanation is more consistent with our findings; given the spatial distribution of pollutant concentrations in Atlanta, a unit increase at the central monitor corresponds to a smaller increase in the overall pollutant levels within the airshed than does a unit increase in the average of the measurements. Assuming that the (log of the) health effect due to air pollution is proportional to the total amount of pollution within the airshed, it is not surprising that the rate ratio on a per unit basis would be lowest for the central monitor measurements. Scaling the rate ratios to an IQR increase is one way to compensate for these differences, although doing so has the disadvantage of tying the interpretation of the rate ratio to the distribution of the pollutant concentrations specific to the study.

## Conclusions

We suggest that the pollutant metric selected for use in a time-series study or health benefits assessment should be based on the research or policy question of interest. Given our results, we expect that the choice of metric could meaningfully impact the estimated health benefits for a reduction in primary pollutants, whereas the selected pollutant metric will likely have a negligible impact on the estimated health benefits for spatially homogeneous pollutants. Although we have focused primarily on the consequences that the choice of metric has for health benefits analyses, investigators may also want to consider this issue when conducting a meta-analysis, as the distribution of results from studies that utilize central monitoring station measurements could be quite different from the distribution of results from studies that average measurements across monitoring stations. Related issues also arise in multi-city studies, as city-to-city heterogeneity in the pollutant effects (per unit increase) might be due in part to monitor siting. Investigating the sensitivity of the estimates of between-city heterogeneity in multi-city studies to changes in the method of characterizing pollutant levels within the airshed could prove helpful in verifying that the apparent heterogeneity is not a consequence of monitor siting.

## List of abbreviations

95%CI: 95% confidence interval; EPA: Environmental Protection Agency; ICD-9: International Classification of Diseases, 9^th ^revision; IQR: interquartile range; NAAQS: National Ambient Air Quality Standards; PM_10_: particulate matter less than 10 microns in diameter; PM_2.5_: particulate matter less than 2.5 microns in diameter; ppb: parts per billion; ppm: parts per million; SOPHIA: Study of Particles and Health in Atlanta; μg/m^3^: micrograms per cubic meter.

## Competing interests

The authors declare that they have no competing interests.

## Authors' contributions

MJS prepared the manuscript. LAD performed the statistical analyses. JAM developed the air pollutant metrics. MK, WDF, AW, and PET helped to conceptualize the study and define the goals for the analysis. All authors participated in the interpretation of results and read and approved the final manuscript.
